# Predictors of Acute and Chronic Undernutrition among 10-19 Year Olds using World Health Organization Growth References: A Cross-sectional Study from Central India

**DOI:** 10.3126/nje.v14i3.55824

**Published:** 2025-07-27

**Authors:** Surya Bali, Revadi Gouroumourty

**Affiliations:** 1 Department of Community and Family Medicine, AIIMS Bhopal, India; 2 Department of Community Medicine, Sri Manakula Vinayagar Medical College, Puducherry, India

**Keywords:** Adolescence, Stunting, Undernutrition, Malnutrition, Anemia

## Abstract

**Background:**

Adolescence, a period of rapid growth and development with biological and physiological changes, is influenced by socioeconomic, cultural, and behavioural factors. There is a dearth of data regarding undernutrition in 10-19 year olds in India. The study's objective was to estimate the prevalence of thinness and stunting in adolescents using World Health Organization (WHO) references and its determinants.

**Methods:**

This cross-sectional study represents the baseline of an adolescent health survey (November 2017 to March 2018) of 3,213 adolescents. Participants were selected using a multi-stage stratified random sampling technique from six districts of Madhya Pradesh. Data were collected by field investigators through a paperless real-time method and analyzed using R and the WHO anthro plus analyzer.

**Results:**

The prevalence of thinness and stunting were 17.1% and 23.3% respectively. Thinness was profound in early adolescents and stunting in late adolescents. While adolescent boys showed a higher prevalence of thinness, girls showed a higher stunting burden. Negative binomial mixed model regression revealed that early adolescents and boys were more likely to develop thinness. Similarly, the likelihood of stunting was higher among late adolescents, girls, and adolescents belonging to categories other than general. Fathers’ education was protective against stunting irrespective of whether they were above or below primary education.

**Conclusion:**

Screening of all adolescents may be undertaken using WHO references in the national surveys for planning interventions targeted to malnourished adolescents to prevent micronutrient deficiencies and intergenerational consequences.

## Introduction

Adolescence is a period of rapid growth and development that is subjected to constant biological and psychological changes often shaped by socioeconomic and cultural factors [1]. Adolescents require a well-balanced diet that includes all nutrients (carbohydrates, proteins, fats, vitamins, and minerals) in appropriate proportions to maintain good health and well-being [2]. Focusing on their well-being can contribute to the epidemiological transition by reducing the mortality and fertility burden, leading to a shift in both the population structure and the predominant pattern of diseases [1].

Malnutrition refers to deficiencies, excesses, or imbalances in a person’s intake of energy and/or nutrients [3]. Chronic undernutrition in adolescents is defined by the WHO in terms of stunting, whereas acute undernutrition is defined as thinness [4]. The WHO 2007 reference standard was used rather than the adult cut-off, as the latter overestimates thinness by 2.5-fold in boys and 4-fold in girls [5]. Poor nutrition can lead to delay or failure in achieving maturation, with a stunted linear growth perpetuating the cycle of poverty and intergenerational undernutrition [5]. The consequences of long-term undernutrition leads to poor cognition and educational performance, low adult wages, lost productivity, and risk of frequent infections [6,7].

In India’s National Family Health Survey 2019-2021 (NFHS), the estimates of acute and chronic undernutrition were limited to children under five at the national level. The Comprehensive National Nutritional Survey conducted in 2016-2018 on 1,200 adolescents from Madhya Pradesh found that the burden of stunting was 29.9% and thinness was 32.3% [7]. Hence, this study aimed to robustly estimate the prevalence of acute and chronic undernutrition in adolescents of selected districts of Madhya Pradesh using WHO references 2007 and its determinants.

## Methodology

### Study Design and Participants

This was a cross-sectional study based on a baseline adolescent health survey conducted in six districts of Madhya Pradesh, namely Shahdol, Anuppur, Jhabua, Dhar, Satna, and Rewa, between November 2017 and March 2018. The study participants were 10-19 year olds. The participants were selected using a multistage stratified random sampling technique, first from six districts of Madhya. The second stage involved randomly selecting three administrative blocks from each of the six districts (a total of 18 blocks). The third stage of stratification was the selection of seven villages/wards from each of these 18 blocks based on the sampling frame of villages (rural) and wards (urban) in the 2011 Census list of villages, using systematic sampling. Finally, 26 adolescents (13 males and 13 females) were randomly selected from each village/ward.

### Data Collection

Field investigators approached households in the villages, and any one adolescent person (boy or girl) from each house, if present, was asked to voluntarily participate in the study. Parental consent was obtained from children aged < 14 years.

### Questionnaire design and validation

Real-time data collection of the pre-tested and pre-validated questionnaires was hosted through a digital cloud server by the field investigators. Each participant was tested for haemoglobin (capillary) using a Hemocue 200 machine, and a cut-off of 12 gm/dl or higher was considered non-anemic [8]. Their corresponding anthropometric measurements of height and weight were also obtained by trained field investigators, with the reading rounded off to the first decimal place. The measurements were regularly checked by a field supervisor.

### Inclusion and Exclusion Criteria

Adolescents already enrolled in the SAATHIYA programme (generating demand for adolescent health services through peer education) were excluded. If one adolescent from a household was included, the other adolescents from the same household were excluded.

### Sample size calculation

The sample size was calculated using Open epi.com with a minimal prevalence of hypertension of 8% [9], 95% confidence interval, and 15% relative error, that is, 1.23 absolute precision error with a design effect of 1.5. The total sample size was 2,862; considering a non-response rate of 15%, a sample size of 3,213 was finalized.

### Outcome variables

Thinness was defined as BMI for age Z-score less than -2 standard deviation from the median reference.

Severe thinness: BMI for age Z score less than -3 standard deviation from the median reference.

Stunting: Height for age Z-score less than -2 standard deviations from the median references.

Severe stunting – height for age Z score less than -3 standard deviation from the median reference.

### Explanatory variables

Sociodemographic characteristics included age, gender, religion, caste, location, district, educational status of the adolescent, socioeconomic characteristics, and marital status of the adolescents. Family level characteristics: living status of the parents, mother’s education, father’s education, mother’s occupation, father’s occupation, and type of family. Behavioral characteristics: Frequency of skipping meals, frequency of physical activity, tobacco consumption, alcohol consumption, and drug intake.

### Ethical considerations

Ethical approval was obtained from the Institutional Human Ethics Committee (IHEC-LOP/2017/EF0069, AIIMS, Bhopal). Written consent from parents of participants below 14 years of age was obtained from each adolescent before including them in the survey.

### Data management and statistical analysis

The data sheet was downloaded in Microsoft Excel, cleaned, coded, and analyzed using WHO Anthro plus software and R software version 4.2.1. Nominal or categorical variables were summarized as proportions, and continuous variables were summarized as mean and SD. Prevalence of thinness and stunting was reported as percentage with 95% confidence interval. Negative binomial mixed model regression was used to calculate the adjusted prevalence ratio in order to avoid overestimating the odds ratio where the prevalence of thinness and stunting were high [10]. Statistical significance was set at P < 0.05.

## Results

The overall prevalence of thinness and severe thinness was 564 (17.1%) with 95% CI (15.8,18.4) and 114 (3.8%) with 95% CI (2.8,4.1), respectively, as per the WHO standards. The overall prevalence of thinness was higher in boys 370 (23%) with (95% CI 21.1,25.3) than girls 194 (12%) with (95% CI 9.7,12.7). Also, the overall prevalence of severe thinness was higher in boys 76 (4.8%) with (95% CI 3.9,6) as compared to girls 38 (2.3%) with (95% CI 1.4,2.7). District wise prevalence of stunting was higher in Anuppur 143(19%), Shahdol 133(17.7%), Satna and Rewa with 130(17.3%) individually, Jhabua 128(17%) and Dhar 87(11.6%).

**Figure 1:**
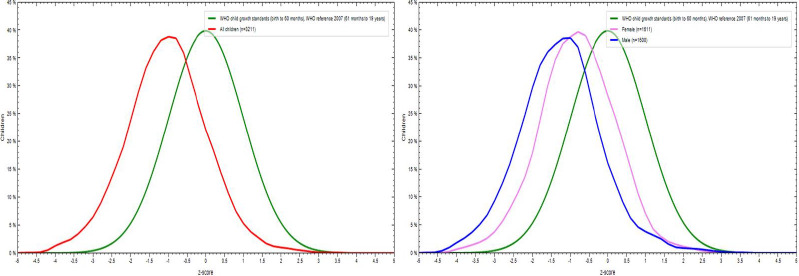
Comparison of distribution curve of BMI for age Z score of adolescents from Central India (N=3213) against the WHO growth reference curve stratified by sex

Figure 1 shows the age-specific BMI (body mass index) of our sample against the WHO reference curve. A shift towards the left (red) with a mean z-score of -1.04, compared to the mean z-score of 0 (green) of the WHO standards, was observed. When stratified by gender, it was found that the mean Z-score of boys (blue) -1.25 had a profound shift towards the left as compared to girls (purple) with a mean Z-score of -0.82.

The overall prevalence of stunting and severe stunting was 751 (23.3%) with (95% CI 21.1,24.8) and 118(3.7%) with (95% CI 3.1,4.1) as per WHO standards. The overall prevalence of stunting was higher in girls (n=1613) at 28% with (95% CI 24.8,29.1) than boys 19% with (95% CI 17.8,21.7). Similarly, the prevalence of severe stunting was slightly higher in girls at 4% with (95% CI 3,4.9) than boys 58 3.6% with (95% CI 2.7,4.6). District wise prevalence of stunting was higher in Dhar 123(21.8%), Jhabua 107(19%), Satna 99(17.6%), Anuppur with 80(14.2%), Rewa 79(14%) and Shahdol 76(13.5%).

The mean Z-score of BMI for age and height for age across younger and older adolescents (10-14 vs.15-19 years) stratified by gender and their corresponding prevalence of thinness, stunting, thinness, and severe stunting are given in Tables 1 and 2.

**Figure 2:**
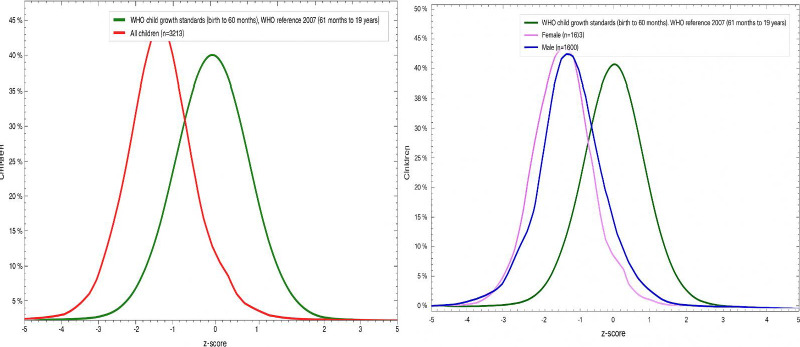
Comparison of distribution curve of height for age Z score of adolescents from Central India (N=3213) against the WHO growth reference curve stratified by sex

Figure 2 shows a comparison of our study’s growth curve with the WHO growth references. A shift towards the left (red), with a mean z-score of -1.36 was observed as compared to the mean z-score of 0 (green) of WHO standards, indicating the burden of chronic undernutrition. When stratified by gender, it was found that the mean Z score of girls (purple) with -1.48 had a profound left shift compared to boys (blue) with -1.24.

Regression analysis of predictors of thinness:

For the thinness model, variables with p-values ≤ 0.20 from table 3 and 4, namely age, sex, education level of the adolescent, socioeconomic status, type of family, mother’s occupation father’s occupation, frequency of skip meals, and frequency of physical activity were adjusted for the confounders in the model (Table 5). The negative binomial mixed model to predict thinness in Table 5 seemed to be a good fit using the Hosmer-Lemeshow test (chi square 11.005, df 8, P 0.201) with low collinearity. Table 5 shows that the likelihood of thinness was more in early adolescents (aPR: 1.56, P 0.002) and adolescent boys (aPR: 1.80, P < 0.001).

Regression analysis of predictors of stunting:

For the stunting model, all variables with P value ≤ 0.20 namely age, sex, caste, educational status, location, socioeconomic status, marital status, type of family, mother’s education, father’s education, mother’s occupation, father’s occupation, and frequency of physical activity (Tables 3 and 4), were considered and adjusted for confounders.

The negative binomial mixed model in Table 6 seemed to be a good fit using the Hosmer-Lemeshow test (chi square 8.594, df 8, P 0.378) with low collinearity. The likelihood of stunting was more likely among late adolescents (aPR: 1.33, P = 0.012), adolescent girls (aPR: 1.36, P < 0.001), those belonging to the scheduled tribe (aPR: 1.96, P <0.001), scheduled caste (aPR: 1.90, P <0.001), and other backward class (aPR: 1.83, P <0.001). Fathers’ education was found to be protective against stunting, irrespective of whether they were educated above or below primary education.

Undernutrition and anaemia:

In total, 1715(53.4%) adolescents were anemic, of which 341 (20%) were thin and 417 (24%) were stunted. Those who were thin were at 1.23 times more likely to be anemic than those who were not thin (95 CI 1.18, 1.71) (P <0.001).

Co-existence of thinness and stunting: 142 (4.4%) adolescents had stunting coexisting with thinness in our study, with 5.8% in boys and 3.1% in girls.

## Discussion

These findings are discussed in the following three sections.

### Burden of acute and chronic undernutrition

Our study found the prevalence of thinness to be 17.1% among 3213 adolescents in the 10-19 years group. This burden of thinness was less than the 32.3% reported by the CNNS survey among 10–19 year-olds in Madhya Pradesh (MP) [7], and the overall 24.4% in India. Similar to MP, all states except states in northeastern India documented a prevalence of thinness of less than 17.1% in the age group of 10-19[7]. The prevalence among 15-19 years adolescents was 8.2%, which was lower than the findings of Bhargava et al. of 12.5% in MP [5] and 10% in India from the NHFS 4 (2015-2016) survey among the same age group.

Similarly, the prevalence of stunting was 23.3% in our study, which was lower than the 29.9% reported in MP and 27.4% in India [7]. Similar to MP, all the states except Jammu and Kashmir, Delhi, Uttarakhand, Punjab, Haryana, Rajasthan, Himachal Pradesh, Goa, Telangana, Tamil Nadu and Kerala documented a prevalence less than 23.3% in adolescents [7]. The prevalence in 15-19 years adolescents was 15%, which was less than 26.9% in MP and 34.1% in India [5]. However, the overall prevalence of stunting is lower than that in Bangladesh [10], Malaysia [11], and African countries such as Ethiopia [12].

The difference of 17% in thinness estimate from our study to that of 24.4% in India and 32.3% in MP from the CNNS survey might be due to the underestimation of thinness by the stunting factor per se [5]. The severe thinness and stunting estimates were found to be 3.8% and 3.7% in our study, which was higher than the 1.7% of thinness and lower than the 6.5% of stunting reported in NFHS 4. This was due to the predominant 15-19 years enrolment in the NFHS, where late adolescence was predisposed to stunting [5]. Approximately 4.4% of adolescents in our study had both stunting and thinness, with 5.8% in boys and 3.1% in girls. This pattern of slightly higher predisposition in boys was comparable with the findings of Kumar et al., who reported concurrent stunting and thinness at 7.8% [13].

### Predictors of thinness

It was found that the likelihood of thinness was higher in boys in our study, similar to a study from South Asia[14] which showed higher estimates of 28.6% in boys and 20.3% in girls. In addition, developing countries such as Indonesia [15] documented 11% thinness in boys and 5% in girls and Nepal [16], with 37.8% in boys and 26.2% in girls. Boys being predisposed to develop thinness compared to girls is consistent with the 2016-2018 CNNS survey (29.9% boys vs. 18.9% girls), where being female was protective against thinness [7]. Second, the prevalence or likelihood of thinness was more profound in early adolescence than in late adolescence in the present study. Study by Kumar et al. in Bihar and Uttar Pradesh [13] and by Gebregyorgis et al. in Ethiopia [12] had found a higher prevalence in boys especially among the early adolescence group. This finding of predisposition in early adolescence has been documented in the CNNS survey where the 15-19 years age group was protective for developing thinness than the 10-14 years [7]. Predisposition of thinness in boys was considered to be due to increased height gain among boys compared to girls [7,12] along with higher caloric deficiencies in the early adolescence stage [17]. The other findings included decreased prevalence of thinness and stunting among the adolescents with irregular physical activity due to impending overweight or obesity.

### Predictors of Stunting

The height attained in adulthood is a combination of pre-pubertal height and the pubertal growth spurt [18] which adds 8.3 cm/year in girls and 9.5 cm/year in boys following additional nutrition [19]. The high levels of stunting could be secondary to the persistence of pre-pubertal deficit from under-five malnutrition [5]. In contrast to the predictors of thinness in our study, those in the late adolescent stage, especially girls, had a higher burden of stunting, similar to the NHFS 4 analysis of Bhargavi et al. [5]and Kumar et al. [13] in Bihar and Uttar Pradesh. However, the opposite was observed among boys in Nepal [16]. Late adolescence and girls were predisposed to higher risk of stunting as compared to boys and early adolescence corroborating with the findings of Pandurangi et al. [7]. The risk of stunting was found to increase with age and was considered attributable to chronic factors such as food security, education, gender disparities and women’s health [20]. In addition, the prevalence of thinness and stunting was higher in adolescents with less than a high school education in our study. This was also reported in the studies by Kumar et al. [13] and Deshmukh et al. [21], where lower educated adolescents were found to be more stunted and thinner. The adolescents belonging to the general category were protective from stunting in our study, which could be due to better socioeconomic status among them. In our study, the education status of the mother with no schooling showed higher predisposition to chronic undernutrition in their children similar to Pandurangi et al. [7]. This might be attributed to the awareness received during their academic courses [22], which might have affected the nutritional status of the children while preparing food and feeding. On the contrary, fathers’ education was protective against stunting, which has not been reported in any studies. They were primarily indulged in agriculture, small businesses, laborers, and private jobs compared to those who were unemployed, which added to their family income and food security, thereby being protective for stunting. In addition, those belonging to the nuclear family were more likely to be stunted, although not a predictor, which was discordant with the findings of a study in Ethiopia [12]. It was believed that extended families provide social care and resources such as food and health care during the absence of their parents [23–25], thereby configuring protection in the long run. This may also explain the protective nature of unmarried adolescents against stunting. Similarly, alcohol intake was protective against stunting in our study, which was similar to the findings of Naude et al. [26] and Wannamethee et al. [27]. Alcohol intake leads to concurrent food intake, which leads to increased total energy consumption and increases the risk of overweight and obesity rather than undernutrition. Few studies from India [28] and Ghana [29] reported anemia and undernutrition separately rather than the association shown in our study.

### Strengths

Our study was community-based and included a large sample representative of the districts; hence, the results can be generalized. Also, 10-19 years were enrolled in our study, in contrast to the study by Bhargava et al., that comprised 15-19 years olds only. We were able to study micronutrient deficiencies such as anemia with undernutrition and concurrent stunting with thinness.

### Limitation of the study

Few determinants, such as comprehensive socioeconomic profile, caloric assessment, employment status of adolescents, parental height, menstruation, history of previous illness, and beneficiaries of the health program were not explored. Recall bias cannot be excluded for behavioral parameters, and reporting bias is inevitable because of self-reporting.

## Conclusion

The thinness and stunting indicators have not been explored robustly in the Indian scenario as a public health problem due to the lack of nationally representative data for 10-19 years. Community-level screening using WHO growth references and the development of indicators is required to identify high-focus districts and compare the progress of nutritional interventions over time.

## Future scope of the study

Targeted health education activities (dietary habits) in high-need districts, especially for 10-16 years adolescents including their parents and school dropouts, should be undertaken. Screening and monitoring growth of adolescents might be done using BMI for age (Z score) and height for age Z score to identify high-risk individuals and areas on a massive scale.

## What is already known about the topic

The burden of malnutrition and its determinants among 15–19 year adolescents from the NFHS 5 survey and those of 10–19-year-olds from the CNNS survey.

What this study adds:

- Burden of undernutrition among 10–19 year olds from a representative sample of Madhya Pradesh State, India.
- The burden and association of micronutrient deficiency, such as anemia and malnutrition
- The prevalence of double burden of malnutrition, such as concurrent stunting with thinness in 10–19 year olds.

## Figures and Tables

**Table 1:** Summary table of BMI for age Z scores for different age groups of 10-19 year olds from Central India, stratified by sex (N=3213)

Age groups		N	BMI-for-age					
Years	Months		Mean Z score	% < -3SD	% < -2SD	% >+1SD	% >+2SD	% > +3SD
**Total (5-19)**	(61-228)	3213	-1.04	3.4	17.1	3	0.6	0.1
**Total (10-14)**	(120-179)	1343	-1.03	4	19.1	4.4	1.3	0.1
**Total (15-19)**	(180-228)	1870	-1.04	2.9	15.6	2	0.2	0.1
**Age groups**	N		BMI for age Z scores (males)					
**Total (5-19)**	(61-228)	1600	-1.25	4.9	23.1	2.8	0.7	0.1
**Total (10-14)**	(120-179)	709	-1.19	5.4	23.1	4.5	1.3	0.1
**Total (15-19)**	(180-228)	891	-1.3	4.5	23.1	1.5	0.2	0
**Age groups**	N		BMI for age Z scores (females)					
**Total (5-19)**	(61-228)	1613	-0.82	1.9	11.1	3.2	0.6	0.1
**Total (10-14)**	(120-179)	634	-0.86	2.5	14.7	4.3	1.3	0
**Total (15-19)**	(180-228)	979	-0.8	1.5	8.8	2.5	0.1	0.1

**Table 2:** Summary table of Height for age Z scores for different age groups of 10-19 year olds from Central India, stratified by sex (N=3213)

Age groups		N	Height-for-age		
Years	Months		% < -3SD	% < -2SD	Mean Z score
**Total (5-19)**	(61-228)	3213	3.7	23.3	-1.36
**Total (10-14)**	(120-179)	1343	3	15.6	-1.09
**Total (15-19)**	(180-228)	1870	4.2	28.9	-1.56
**Age groups**	N		Height for age Z scores (males)		
**Total (5-19)**	(61-228)	1600	3.8	19.7	-1.24
**Total (10-14)**	(120-179)	709	3	13.5	-0.96
**Total (15-19)**	(180-228)	891	4.5	24.6	-1.47
**Age groups**	N		BMI for age Z scores (females)		
**Total (5-19)**	(61-228)	1613	3.5	27	-1.48
**Total (10-14)**	(120-179)	634	3	17.8	-1.23
**Total (15-19)**	(180-228)	979	3.9	32.9	-1.64

**Table 3:** Results of univariate regression to determine the socio demographic characteristics predicting thinness and stunting among the adolescents in Central India (N=3213)

Thinness				Stunting					
Characteristics	Overall N = 3,213^1^	No N = 2,649^1^	Yes N = 564^1^	Unadjusted PR^2^ (95%CI)	p-value	No N = 2,462^1^	Yes N = 751^1^	Unadjusted PR^2^ (95%CI)	p-value
**Age**									
**Late (17-19)**	950(29.6)	819(30.9)	131(23.2)	-		678(27.5)	272(36.2)	-	
**Middle (14-16)**	1,350(42)	1,127(42.5)	223(39.5)	1.20(0.98,1.48)	0.083	1,048(42.6)	302(40.2)	0.78(0.66,0.92)	0.004
**Early (10-13)**	913(28.4)	703(26.5)	210(37.2)	1.64(1.33,2.03)	<0.001	736(29.9)	177(23.6)	0.68(0.56, 0.83)	<0.001
**Sex**									
**Female**	1,613(50.2)	1,419(53.6)	194(34.4)	-		1,167(47.4)	446(59.4)	-	
**Male**	1,600(49.8)	1,230(46.4)	370(65.6)	1.92(1.62,2.28)	<0.001	1,295(52.6)	305(40.6)	0.69(0.60, 0.80)	<0.001
**Caste categories**									
**Scheduled Tribe**	1,317(41)	1,071(40.4)	246(43.6)	-		973(39.5)	344(45.8)	-	
**Scheduled Caste**	486(15.1)	410(15.5)	76(13.5)	0.84(0.65,1.10)	0.2	355(14.4)	131(17.4)	0.98(0.80, 1.21)	0.9
**Other Backward class**	1,046(32.6)	864(32.6)	182(32.3)	0.93(0.76,1.14)	0.5	813(33)	233(31)	0.82(0.69, 0.98)	0.030
**General**	364(11.3)	304(11.5)	60(10.6)	0.89(0.67,1.18)	0.4	321(13)	43(5.7)	0.42(0.31, 0.58)	<0.001
**Religion**									
**Hindu**	2,982(92.8)	2,449(92.4)	533(94.5)	-		2,289(93)	693(92.3)	-	
Others*	82(2.6)	70(2.6)	12(2.1)	0.71(0.40,1.28)	0.3	64(2.6)	18(2.4)	1.05(0.67, 1.64)	0.8
**Muslim**	149(4.6)	130(4.9)	19(3.4)		0.3	109(4.4)	40(5.3)	1.12(0.81, 1.54)	0.5
**Location**									
**Rural**	2,539(79)	2,090(78.9)	449(79.6)	-		1,927(78.3)	612(81.5)	-	
**Urban**	674(21)	559(21.1)	115(20.4)	0.94(0.76,1.15)	0.5	535(21.7)	139(18.5)	0.87(0.72, 1.04)	0.12
**Education level of the adolescents**									
**High School**	544(16.9)	466(17.6)	78(13.8)	-		418(17)	126(16.8)	-	
**Intermediate**	2,403(74.8)	1,978(74.7)	425(75.4)	1.22(0.96,1.54)	0.10	1,825(74.1)	578(77)	1.04(0.87, 1.26)	0.7
**Up to Primary education**	266(8.3)	205(7.7)	61(10.8)	1.51(1.10,2.08)	0.012	219(8.9)	47(6.3)	0.77(0.56, 1.06)	0.10
**Socio economic status of the adolescents**									
**Below poverty line**	2,176(67.7)	1,778(67.1)	398(70.6)	-		1,648(66.9)	528(70.3)	-	
**Above poverty line**	1,037(32.3)	871(32.9)	166(29.4)	0.86(0.72,1.03)	0.11	814(33.1)	223(29.7)	0.89(0.76, 1.04)	0.2

**Table 4:** Results of univariate regression analysis to determine the family and behavioural characteristics predicting thinness and stunting among adolescents in Central India

Thinness				Stunting					
Characteristics	Overall N = 3,213^1^	No N = 2,649^1^	Yes N = 564^1^	Unadjusted PR^2^(95%CI)	p-value^2^	No N = 2,462^1^	Yes N = 751^1^	Unadjusted PR^2^(95%CI)	p-value^2^
**A. Family characteristics**									
**Family type**									
**Nuclear**	2,225(69.2)	1,856(70.1)	369(65.4)	-		1,683(68.4)	542(72.2)	-	
**Non-nuclear**	988(30.8)	793(29.9)	195(34.6)	1.16(0.97,1.38)	0.10	779(31.6)	209(27.8)	0.88(0.75, 1.03)	0.10
**Living status of the parents**									
**Both Alive**	2,948(91.8)	2,430(91.7)	518(91.8)	-		2,267(92.1)	681(90.7)	-	
**Father Dead**	180(5.6)	145(5.5)	35(6.2)	1.10(0.79,1.53)	0.6	133(5.4)	47(6.3)	1.14 (0.86, 1.51)	0.4
**Mother Dead**	67(2.1)	57(2.2)	10(1.8)	0.85(0.50,1.43)	0.5	51(2.1)	16(2.1)	1.04(0.67, 1.61)	0.9
**Both Dead**	18(0.6)	17(0.6)	1(0.2)	0.33(0.15,0.71)	0.004	11(0.4)	7(0.9)	1.65(0.89, 3.07)	0.11
**Marital status**									
**Unmarried**	3,174(98.8)	2,615(98.7)	559(99.1)	-		2,436(98.9)	738(98.3)	-	
**Married**	39(1.2)	34(1.3)	5(0.9)	0.68(0.34,1.39)	0.3	26(1.1)	13(1.7)	0.43(0.87, 2.36)	0.2
Mothers’ Education*									
**High School**	325(10.4)	271(10.5)	54(9.8)	-		274(11.4)	51(7)	-	
**Intermediate**	519(16.6)	424(16.5)	95(17.2)	1.08(0.78,1.50)	0.7	423(17.6)	96(13.2)	1.20(0.88, 1.63)	0.3
**Primary**	831(26.6)	682(26.5)	149(26.9)	1.03(0.76,1.41)	0.8	663(27.6)	168(23.1)	1.33(1.00, 1.76)	0.049
**Illiterate**	1,453(46.5)	1,198(46.5)	255(46.1)	1(0.75, 1.35)	>0.9	1,040(43.3)	413(56.7)	1.86(1.43, 2.42)	<0.001
**Fathers’ Education^**									
**High School**	603(20)	492(19.8)	111(21)	-		411(17.7)	192(27.5)	-	
**Intermediate**	727(24.1)	589(23.6)	138(26.1)	1.04(0.81,1.3)	0.8	550(23.7)	177(25.4)	0.76(0.62, 0.93)	0.009
**Primary**	942(31.2)	780(31.4)	162(30.7)	0.95(0.74,1.21)	0.7	753(32.5)	189(27.1)	0.62(0.51, 0.76)	<0.001
**Illiterate**	743(24.6)	626(25.2)	117(22.2)	0.88(0.68,1.14)	0.3	604(26.1)	139(20)	0.58(0.46, 0.72)	<0.001
Mothers’ Occupation*									
**Unemployed**	1,438(46)	1,206(46.8)	232(42)	-		1,126(69.1)	312(66)	-	
**Employed**	1690(54)	1369(53.2)	321(58)	1.13(0.95,1.34)	0.2	503(30.9)	161(34)	1.15(0.99, 1.33)	0.064
**Fathers’ Occupation^**									
**Unemployed**	39(1.3)	29(1.4)	10(1.9)	-		27(4.1)	12(5.6)	-	
**Employed**	2976(98.7)	2058(98.6)	518(98.1)	0.66(0.36,1.18)	0.2	637(95.9)	204(94.4)	0.75(0.51, 1.10)	0.14
**B. Behavioural characteristics**									
**History of skip meals^$^**									
**Never**	1268(41)	1018(39.9)	250(46.3)	-		972(41.2)	296(40.5)	-	
**Occasionally**	1824(59)	1534(60.1)	290(53.7)	0.81(0.68,0.95)	0.012	1389(58.8)	435(59.5)	1.02(0.88, 1.19)	0.7
**Physical activity**									
**Regular**	1,864(58)	1,488(56.2)	376(66.7)	-		1,476(60)	388(51.7)	-	
**Irregular**	1,349(42)	1,161(43.8)	188(33.3)	0.70(0.59,0.83)	<0.001	986(40)	363(48.3)	1.29(1.12, 1.49)	<0.001
**Alcohol consumption**	115(3.6)	102(3.9)	13(2.3)	0.65(0.37,1.13)	0.23	95(3.9)	20(2.7)	0.72(0.48, 1.07)	0.3
**Tobacco consumption**	439(13.7)	355(13.4)	84(14.9)	1.14(0.91,1.43)	0.3	346(14.1)	93(12.4)	0.88(0.71, 1.09)	0.3
**Usage of drugs**	41(1.3)	32(1.2)	9(1.6)	1.13(0.77,2.29)	0.3	36(1.5)	5(0.7)	0.51(0.30, 0.88)	0.23

^1^Frequency (%),

^2^Pearson's Chi-squared test; Fisher's exact test and

*85 data missing,

^- 198 data missing and $-121 data missing

**Table 5:** Results of multivariate regression depicting the socio demographic, family and behavioural predictors of thinness amongst adolescents in Central India (N=564)

Characteristic	aPR^1^	95% CI^1^	p-value
**A. Socio demographic**			
**Age**			
**Late (17-19)**	—	—	
**Middle (14-16)**	1.20	0.93, 1.55	0.2
** Early (10-13)**	1.56	1.18, 2.06	0.002
**Sex**			
**Female**	—	—	
**Male**	1.80	1.48, 2.20	<0.001
**Education level of the adolescents**			
**High School**	—	—	
**Intermediate**	0.95	0.71, 1.27	0.7
**Up to Primary education**	0.95	0.63, 1.43	0.8
**Socio economic status of the adolescents**			
**Below poverty line**	—	—	
**Above poverty line**	0.84	0.69, 1.02	0.080
**B. Family** **characteristics**			
**Family type**			
**Nuclear**	—	—	
**Non-nuclear**	1.19	0.98, 1.43	0.072
**Mothers’ Occupation**			
** Unemployed**	—	—	
**Employed**	1.03	0.86, 1.24	0.7
**Fathers’ Occupation**			
**Unemployed**	—	—	
**Employed**	0.70	0.36, 1.37	0.3
**C. Behavioural characteristics**			
**History of skip meals**			
**Never**	—	—	
**Occasionally**	0.91	0.76, 1.09	0.3
**Physical activity**			
** Regular**	—	—	
** Irregular**	0.95	0.78, 1.16	0.6

**Table 6:** Results of multivariate regression depicting the socio demographic, family and behvioural predictors of stunting among the adolescents (N=751)

Characteristic	aPR^1^	95% CI^1^	p-value
**A. Socio demographic**			
**Age**			
**Early (10-13)**	—	—	
**Middle (14-16)**	1.06	0.87, 1.29	0.6
**Late (17-19)**	1.33	1.06, 1.66	0.012
**Sex**			
**Male**	—	—	
**Female**	1.36	1.15, 1.60	<0.001
**Caste categories**			
**General**	—	—	
**Other Backward class**	1.90	1.32, 2.72	<0.001
**Scheduled Caste**	1.96	1.35, 2.85	<0.001
**Scheduled Tribe**	1.83	1.30, 2.58	<0.001
**Location**			
**Rural**	—	—	
**Urban**	0.89	0.72, 1.09	0.3
**Education level of the adolescents**			
**High School**	—	—	
**Intermediate**	1.05	0.84, 1.31	0.7
**Up to Primary education**	0.73	0.49, 1.09	0.12
**Socio economic status of the adolescents**			
**Below poverty line**	—	—	
**Above poverty line**	1.04	0.88, 1.23	0.7
**B. Family characteristics**			
**Family type**			
**Nuclear**	—	—	
**Non-nuclear**	0.91	0.76, 1.08	0.3
**Marital status**			
**Unmarried**	—	—	
**Married**	1.11	0.61, 2.04	0.7
**Mothers’ Education**			
**High School**	—	—	
**Intermediate**	1.09	0.77, 1.54	0.6
**Primary**	1.09	0.78, 1.51	0.6
**Illiterate**	1.31	0.95, 1.81	0.10
**Fathers’ Education**			
**High School**	—	—	
**Intermediate**	0.83	0.67, 1.03	0.093
**Primary**	0.74	0.59, 0.93	0.009
**Illiterate**	0.73	0.56, 0.94	0.013
**Mothers’ occupation**			
**Homemaker**	—	—	
**Employed**	1.05	0.89, 1.23	0.6
**Fathers’ occupation**			
**Unemployed**	—	—	
**Employed**	0.79	0.45, 1.38	0.4
**C. Behavioural characteristics**			
**Physical activity**			
** Regular**	—	—	
**Irregular**	1.12	0.95, 1.32	0.2

^1^aPR-Adjusted prevalence ratio, CI = Confidence Interval
